# Development and Validation of a Simple-to-Use Nomogram of In-Hospital Heart Failure in Patients with Acute Myocardial Infarction

**DOI:** 10.3390/jcm15010194

**Published:** 2025-12-26

**Authors:** Ou Zhang, Yu Geng, Lei Bi, Jian Jia, Siyuan Li, Haowen Xue, Yintang Wang, Yifei Wang, Ping Zhang

**Affiliations:** 1 Department of Cardiology, Beijing Anzhen Hospital, Capital Medical University, Beijing 100029, China; zho16@163.com; 2 Department of Cardiology, Beijing Tsinghua Changgung Hospital, School of Clinical Medicine, Tsinghua University, Beijing 102218, China; gya02137@btch.edu.cn (Y.G.); bla04035@btch.edu.cn (L.B.); lsya03614@btch.edu.cn (S.L.); zpa00593@btch.edu.cn (P.Z.); 3 Department of Cardiology, Zhungeer Banner Central Hospital, Ordos 010400, China; admin@zhungeerqizxyy.com; 4 Department of Cardiology, College of Arts and Sciences, University of North Carolina at Chapel Hill, Chapel Hill, NC 27599, USA; haowen@unc.edu

**Keywords:** heart failure, acute myocardial infarction, nomogram, risk

## Abstract

**Background**: Patients with acute myocardial infarction (AMI) who experience in-hospital heart failure (HF) would present a higher risk for fatal events. This study aims to develop and validate a simple-to-use diagnostic nomogram to identify high-risk individuals for in-hospital HF in patients with AMI. **Methods**: Using data from CCC-ACS (Improving Care for Cardiovascular Disease in China-Acute Coronary Syndrome) project (2014–2019), this study included 74,697 patients with ST elevation myocardial infarction (STEMI) or non-STEMI (NSTEMI) who admitted within 24 h after symptom onset, without HF, cardiac arrest, or cardiac shock at admission. Independent predictors were identified through univariate logistic regression analyses and least absolute shrinkage and selection operator (LASSO) regression. A nomogram was subsequently constructed based on multivariate logistic regression. The model’s performance was evaluated by its discrimination and calibration, assessed using Harrell’s C-index and calibration curves with Hosmer–Lemeshow goodness-of-fit tests, respectively. **Results**: Six predictors were selected for the final nomogram, including age, heart rate, history of atrial fibrillation, history of chronic obstructive pulmonary disease, history of chronic HF, and history of chronic kidney disease. The nomogram demonstrated a C-index of 0.68 (95% CI: 0.66–0.69) in the training cohort and 0.67 (95% CI: 0.66–0.69) in the validation cohort. The calibration curves of the nomogram showed a strong calibration, as Hosmer–Lemeshow goodness-of-fit tests yielded chi-squares of 11.00 (*p* = 0.21) and 8.48 (*p* = 0.39) for the training and validation cohort, respectively. **Conclusions**: This simple-to-use nomogram for effectively predicting the risk for in-hospital HF may be used as a helpful tool in clinical decision-making during treatment and management in patients with AMI.

## 1. Introduction

Coronary heart disease (CHD) remains a major global health burden and a leading cause of mortality worldwide [1,2]. Acute myocardial infarction (AMI), the most severe manifestation of CHD, is strongly associated with major adverse cardiovascular events (MACEs) and substantial morbidity [3,4]. The pathophysiological process of AMI involves irreversible loss of cardiomyocytes, which frequently progresses to heart failure (HF) or even cardiogenic shock [5,6]. Clinical evidence indicates that the incidence of HF following AMI ranges from 7% to 38% across different trial populations [7]. Particularly concerning is the robust association between HF development and increased in-hospital mortality among AMI patients, with more than half of those experiencing acute decompensated HF succumbing within five years [8,9].

Risk prediction scoring systems have emerged as valuable tools for prognostic assessment in AMI patients, most established models primarily focus on mortality endpoints [10,11,12]. However, this may emphasis overlooks the critical importance of in-hospital HF as an early indicator of poor prognosis. Currently, there is a notable scarcity of readily applicable scoring systems specifically designed to predict in-hospital HF in this vulnerable population. Furthermore, existing prediction models often incorporate numerous variables and require complex calculations, which substantially limits their utility in routine clinical practice [13,14]. This gap underscores the urgent need for a simple, practical risk stratification tool that can be seamlessly integrated into clinical workflows.

To address this unmet clinical need, we conducted the present study to develop and validate a simplified risk prediction model for in-hospital HF in patients with AMI. This tool aims to facilitate early identification of high-risk individuals, enable proactive management strategies, optimize therapeutic interventions, and ultimately improve long-term clinical outcomes in this patient population.

## 2. Materials and Methods

### 2.1. Study Design and Participants

The Improving Care for Cardiovascular Disease in China Acute Coronary Syndrome Project (CCC-ACS) is a prospective, nationwide registry established through a collaboration between the American Heart Association and the Chinese Society of Cardiology, with the overarching aim of enhancing the quality of care for ACS in China. The project was initiated in November 2014 with a cohort of 150 tertiary hospitals, selected to ensure representation from varied geographical and socioeconomic regions. A subsequent expansion in 2017 incorporated 82 secondary and 9 additional tertiary hospitals. The methodology of the CCC-ACS project has been extensively detailed previously [15]. Briefly, patients diagnosed with ACS—encompassing ST-segment elevation myocardial infarction (STEMI), non-STEMI (NSTEMI), and unstable angina—were consecutively recruited on a monthly basis from 241 participating hospitals. Identification was based on the principal discharge diagnosis through a review of inpatient lists, at a rate of 20 to 30 patients per tertiary hospital and 10 to 20 per secondary hospital each month. And the definition of ACS was in accordance with the Chinese Society of Cardiology guidelines for the diagnosis and management of patients with NSTE-ACS [16] and STEMI [17] (see Supplementary Materials).

**Figure 1:**
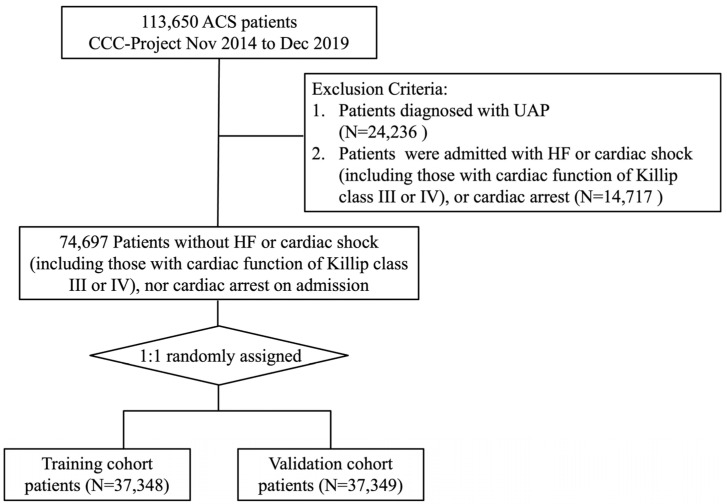
Study flow chart. ACS = acute coronary syndrome; AMI = acute myocardial infarction; CCC = Improving care for cardiovascular disease in China; UAP = unstable angina pectoris.

From the overall population of 113,650 ACS inpatients enrolled between November 2014 and December 2019, a specific cohort for this analysis was derived. The final study population was restricted to 74,697 patients admitted with STEMI or NSTEMI within 24 h of symptom onset. Exclusion criteria comprised the presence of HF, cardiogenic shock (including those with cardiac function of Killip class III or IV), or cardiac arrest upon admission, as delineated in Figure 1. Ethical approval for this study was granted by the Ethics Committee of Beijing Anzhen Hospital, Capital Medical University (Approval No. 2014018), with a waiver for informed consent.

### 2.2. Data Collection and Variables

The collection of clinical information was conducted by trained data abstractors across all participating hospitals, utilizing a centralized electronic data capture platform. Data on patient demographics, medical histories, clinical features upon presentation, acute treatment, and in-hospital outcomes were sourced directly from patient medical documents and compiled in accordance with standardized definitions provided in the project operations manual. And data accuracy and completeness were ensured by the following approaches: online automatic checks for invalid values, face-to-face training workshops, onsite quality control, monitoring of data completeness, and third-party audits.

All data utilized in the present analysis were obtained from the CCC-ACS project database. Medical history was based on patient self-reporting. All the clinical conditions including heart rate (HR), systolic blood pressure (SBP), diastolic pressure (DBP), weight, height, and Killip class were measured and recorded at the time of hospital admission. The administration of guideline-recommended medications, including aspirin, P2Y12 inhibitors, statins, angiotensin-converting enzyme inhibitors (ACEIs) or angiotensin receptor blockers (ARBs), and beta-blockers, was documented within the first 24 h following the initial medical contact. The primary endpoint for this study was the occurrence of in-hospital HF. This outcome was defined as any HF event diagnosed by the treating physician and documented in the patient’s medical record during the hospitalization period, which included cases classified as Killip class II, III, or IV.

### 2.3. Statistical Analyses

The final study population was randomly divided into a training cohort and an internal validation cohort at a 1:1 ratio, resulting in 37,348 patients in the training set and 37,349 in the validation set (Figure 1). For baseline characteristics, Categorical variables were expressed as frequencies and percentages, while continuous variables were presented as means with standard deviations or medians with interquartile ranges, as appropriate. Potential predictors were pre-specified based on clinical relevance, existing literature on HF, and variables showing statistically significant differences between the in-hospital HF and non-HF groups in the training cohort. Meanwhile, in order to build a simple-to-use model, we restricted candidate predictors to those obtainable through non-invasive methods. Variable selection was performed using least absolute shrinkage and selection operator (LASSO) logistic regression model. And predictors to build the final nomogram were selected according to the optimal lambda (λ). λ is the regularization parameter in LASSO, and the optimal value could be obtained from the 10-fold cross-validation. To enhance model parsimony, we selected the value of λ corresponding to the largest penalty within one standard error of the minimum binomial deviance, thereby applying a stricter threshold for variable retention. Collinearity of the selected predictors was detected using variance inflation factor (VIF), with a VIF < 5 considered acceptable.

A multivariate logistic regression model was then developed using the predictors retained by the LASSO procedure. Model performance was evaluated in both the training and validation cohorts. Discrimination was quantified using Harrell’s C-index, and calibration was assessed visually with calibration curves and statistically with the Hosmer–Lemeshow goodness-of-fit test across 10 risk groups. The clinical utility of the prediction model was further examined using decision curve analysis (DCA), with zero indicating no benefit and values > 0 indicating increasing benefit. And Net benefit was calculated as the difference between the true-positive rate and the false-positive rate, weighted by the odds of the risk threshold, and plotted across a range of clinically reasonable probability thresholds. Finally, we constructed a nomogram to visualize the model and a web-based calculator to facilitate individual risk estimation of in-hospital HF. Patients were also categorized by nomogram score quartiles, and the associated odds ratios (ORs) with 95% confidence intervals (CIs) for in-hospital HF were reported.

All analyses were conducted in compliance with the Transparent Reporting of a Multivariable Prediction Model for Individual Prognosis or Diagnosis (TRIPOD) statement (Table S1). Statistical analyses were performed using SAS 9.4, and the nomogram was developed with R 4.0. A two-sided *p*-value < 0.05 was considered statistically significant.

## 3. Results

### 3.1. Baseline Characteristics

A total of 74,697 patients were included in the final analysis, comprising 52,436 (70.19%) with STEMI and 22,261 (29.81%) with NSTEMI. The overall incidence of in-hospital HF was 4.10% (3059 events). In the training cohort (n = 37,348), the mean patient age was 62.31 ± 12.47 years, and 76.98% were male. A total of 1467 patients (3.93%) developed in-hospital HF. Compared to those without HF, patients who developed in-hospital HF were significantly older, more likely to be female, and had a higher HR at admission. They also exhibited a higher prevalence of atrial fibrillation (AF), hypertension, diabetes mellitus (DM), chronic heart failure (CHF), and STEMI diagnosis. These characteristic patterns were consistently observed in the validation cohort (n = 37,349; mean age 62.32 ± 12.46 years; 76.80% male), where 1592 (4.26%) patients developed in-hospital HF, demonstrating similar risk profiles across both datasets (Table 1).

### 3.2. Selection of Predictors for the Nomogram of In-Hospital HF

**Figure 2:**
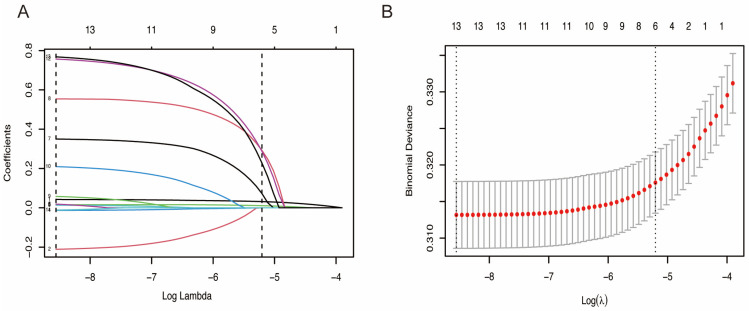
Features selection by LASSO. (**A**) LASSO coefficients profiles (*y*-axis) of the 13 features. The upper *x*-axis shows the average numbers of predictors and the lower *x*-axis shows the log(λ). (**B**) Tenfold cross-validation for tuning parameter selection in the LASSO model.

In the training cohort, statistically significant differences were observed in 13 simple-to-obtain predictors (Table S2), including age, gender, HR, SBP, DBP, and medical histories of AF, hypertension, DM, chronic obstructive pulmonary disease (COPD), CHF, chronic kidney disease (CKD), current smoking status, and previous MI. Using LASSO logistic regression model, 6 predictors were selected to build the final nomogram for predicting in-hospital HF risk in patients with AMI when log (λ) = 0.0055 (Figure 2), and the other 7 factors were excluded for their regression coefficients were compressed to zero. We used VIF to detect the collinearity of these six predictors (Table S3), then calculated the multivariable OR for them (Table 2). Continuous variables including age and HR, and categorical variable including previous AF, previous COPD, previous CHF, and previous CKD. The points assigned to each predictor reflect their relative contribution to the outcome, as quantified by the beta coefficients from the logistic regression equation.

### 3.3. Construction and Validation of Nomogram

**Figure 3:**
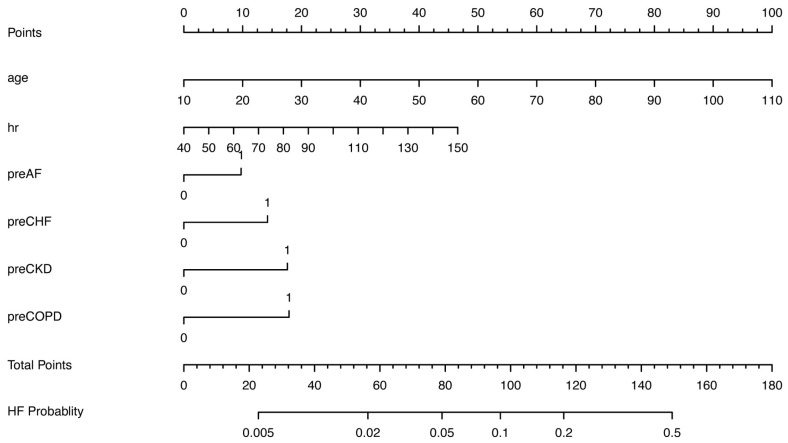
Nomogram predicting HF in patients with AMI. AF = atrial fibrillation; CHF = chronic heart failure; CKD = chronic renal dysfunction; COPD = chronic obstructive pulmonary disease; HR = heart rate.

**Figure 4:**
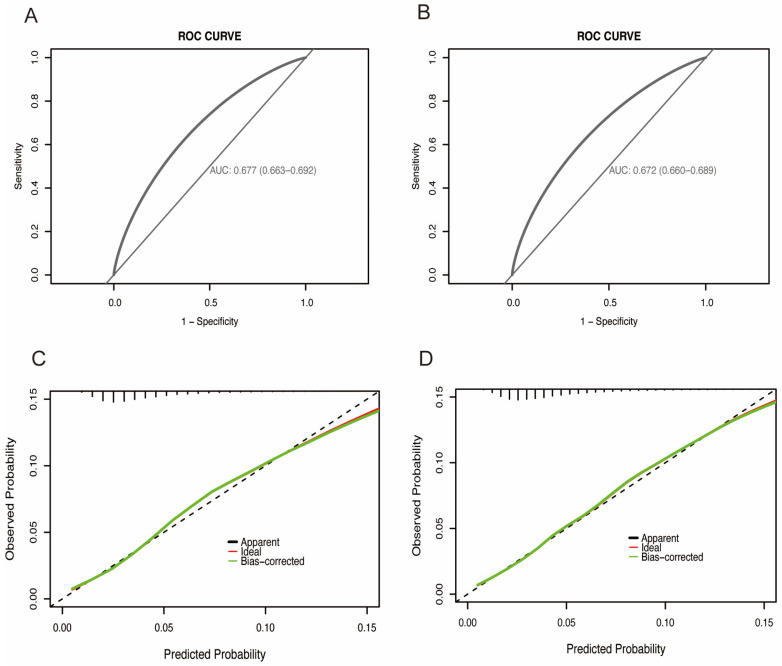
The ROC curve and calibration curves of the HF incidence in training set and internal validation set. (**A**) AUC of the ROC curve in training set. (**B**) AUC of the ROC curve in internal validation set. (**C**) Calibration curves of the HF incidence in training set. (**D**) Calibration curves of the HF incidence in internal validation set.

The six independent predictors identified through variable selection were integrated into a nomogram for estimating the probability of HF (Figure 3). Each predictor was assigned a specific point value, and the cumulative total of these points corresponds to an individual’s risk level, with higher scores indicating greater probability of HF development (Table 3). This simple nomogram demonstrated good discriminative ability for in-hospital HF events, with a Harrell’s C-index of 0.68 (95% CI: 0.66–0.69) in the training cohort and 0.67 (95% CI: 0.66–0.69) in the validation cohort (Figure 4A,B). Calibration performance was also satisfactory: the calibration plot of actual versus predicted probabilities is displayed in Figure 4C,D, and Hosmer–Lemeshow goodness-of-fit tests yielded chi-squares of 11.00 (*p* = 0.20) and 8.48 (*p* = 0.39) for the training and validation cohort, respectively.

**Figure 5:**
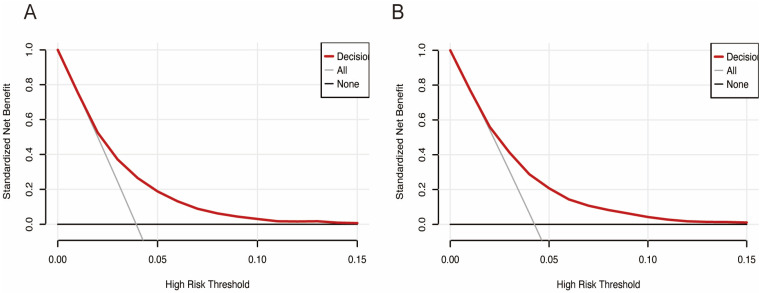
Decision curve analysis (DCA) of the nomogram. (**A**) DCA of the nomogram in training set. (**B**) DCA of the nomogram in validation set.

A DCA was conducted to evaluate the clinical utility of the prediction model (Figure 5). The results demonstrated that when the nomogram-predicted probability of in-hospital HF was below approximately 5%, the model provided greater net benefit compared to both the “treat-all” and “treat-none” strategies. This pattern was consistently observed in both the training and validation datasets, indicating the nomogram’s potential value in clinical decision-making for identifying low-risk patients who might not require intensive intervention.

### 3.4. Construction of Web App to Easily Access the Nomogram

To calculate individual patient scores and corresponding HF probabilities, we made a web-based calculator on basis of the nomogram. The algorithm calculates a patient’s probability of in-hospital HF automatically and enables to identify the high-risk patients, which could facilitate adoption of appropriate treatment measures to these patients early. For example, when a patient has an age of 76 years, HR of 103 bpm, history of AF, CHF, CKD, and COPD, the probability of the in-hospital HF would be 0.47 (95% CI: 0.36–0.59) (Figure S1).

### 3.5. In-Hospital HF Events by Risk Score Categories

**Figure 6:**
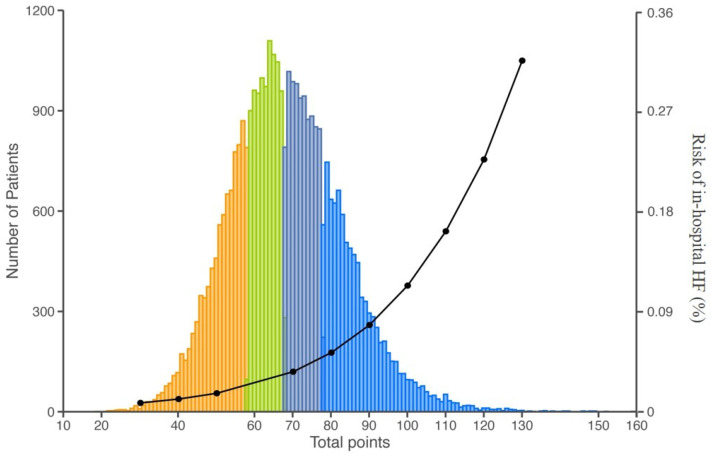
In-hospital HF events among patients with AMI across score quartiles.

Based on the nomogram-derived risk scores, patients were stratified into four distinct risk quartiles: low risk (≤60 points), moderate risk (>60 to ≤70 points), high risk (>70 to ≤80 points), and very high risk (>80 points), in-hospital HF event rates were consistently separated by score quartiles (Table 3 and Figure 6). In-hospital HF events across quartiles were 1.5%, 2.5%, 4.3%, and 9.1% (overall *p* < 0.001), with a particularly pronounced rise observed in the top quartile. Compared with the lowest quartile, the risk of HF was significantly elevated for the second quartile (OR:1.21; 95% CI: 1.12–1.31; *p* < 0.001) and the third-quartile (OR: 1.56; 95% CI:1.44–1.68; *p* < 0.001), and significantly doubled for fourth-quartile (OR: 2.28; 95% CI: 2.13–2.45; *p* < 0.001) patients. Comparable results were also observed in the validation cohort (Table S4).

## 4. Discussion

Based on a large, geographically diverse national cohort, we developed and validated a clinical prediction nomogram for in-hospital HF in patients with AMI. The nomogram incorporates six readily obtainable clinical variables: including age, HR, previous AF, previous CHF, previous COPD, and previous CKD. This nomogram demonstrated good discriminative performance in identifying AMI patients at risk for HF and showed favorable clinical utility on decision curve analysis. With its simple structure and reliance on routinely available parameters, this tool provides clinicians with a practical means for early risk stratification, potentially enabling more timely interventions and improved patient management.

CHD is a well-established primary etiology of HF [18]. When poorly controlled, CHD can progressively lead to HF, significantly complicating clinical management and adversely impacting patient prognosis. AMI, as the most severe type of CHD, frequently results in ischemic cardiomyopathy due to irreversible myocardial loss, thereby serving as a common precursor to HF [5]. Consequently, the early identification of AMI patients at high risk for developing HF is a critical clinical priority. Effective risk stratification is fundamental to guiding therapeutic decision-making and optimizing patient care [14]. Although previous studies have investigated risk factors for HF, early diagnosis remains challenging. Older age, elevated HR at presentation, DM, prior MI, and enrolling MI were found risk factors for in-hospital HF in patients with NSTE-ACS. However, the generalizability of these findings, derived from a clinical trial population, to broader, real-world NSTE-ACS cohorts may be limited. Furthermore, this model was not specifically designed for the AMI population [19]. Tan et al. found that hypertension, age, the total bilirubin, uric acid, urea nitrogen, triglyceride, and total cholesterol levels are associated with HF in patients with CHD. Including these parameters, a nomogram predicting HF in CHD was established and validated [20]. However, AMI is not equivalent to CHD. Its predicting performance in AMI patients was underdetermined. Additionally, parameters involving laboratory examinations may impede rapid, early risk stratification in routine clinical practice.

In our study, only parameters involving medical history and physical examinations were included. All information could be obtained at the first site of access to the AMI patients, making the application of the nomogram and scoring system convenient for clinicians. Our nomogram achieved C-indices of 0.68 and 0.67 in the training and validation cohorts, respectively, indicating modest but clinically useful discriminative ability. While this performance is lower than models incorporating complex variables, it reflects our intentional design to create a practical tool using only six readily available parameters. The model demonstrated strong clinical utility by effectively stratifying patients into distinct risk categories, with in-hospital HF event rates increasing sharply from 1.5% in the low-risk group to 9.1% in the very high-risk group. And decision curve analysis further confirmed its clinical value, showing consistent net benefit across realistic risk thresholds. This suggests that employing our nomogram to intensify monitoring for high-risk individuals could be more beneficial than following a “treat-all” or “treat-none” strategy. It aligns with our purpose to build this study—to provide a practical and immediate risk assessment at the point of care, facilitating early and targeted management for AMI patients susceptible to in-hospital HF.

The observed incidence of in-hospital HF (approximately 4%) was lower than the 7–38% range reported in previous studies. This difference can be largely explained by our exclusion of patients with pre-existing HF, cardiac arrest, or cardiogenic shock (Killip class III–IV) at admission, which defined a lower-risk baseline cohort compared to all-comer AMI populations in other registries. Additionally, potential under-ascertainment of mild HF events due to reliance on physician documentation in medical records may also have contributed to the lower event rate.

Consistent with existing literature, our analysis confirmed several patient characteristics at presentation as significant predictors of HF following AMI. For instance, female sex was associated with a higher risk for heart failure. In a large all-comers AMI survivors’ cohort, females had a higher HF risk [21]. Sex-specific differences in AMI might involve myocardial function, coronary anatomy and physiology, post-AMI care, response to HF medications and differences in medication adherence [22,23,24]. Although sex was not retained in our final nomogram, as its regression coefficient was penalized to zero during LASSO variable selection, its established biological and clinical relevance underscores the complexity of HF risk stratification. We further confirmed that a history of AF significantly increases the likelihood of in-hospital HF in AMI patients, aligning with prior reports linking AF to HF hospitalization in these patients [25]. As commonly known, AF and HF often coincide in clinical practice. Therefore, AF complicating AMI can be a potential preventive and therapeutic target of post-MI HF. Additionally, impaired renal function emerged as a strong predictor of HF risk. While previous studies have highlighted the prognostic impact of renal dysfunction following AMI hospitalization [26], our results specifically demonstrate its direct association with in-hospital HF occurrence. Patients with CKD developed HF at significantly higher rates than those without renal impairment, supporting the value of incorporating baseline CKD status into risk estimation. Similarly, we found that a history of COPD was associated with worse outcomes, consistent with known shared risk factors and overlapping mechanisms—including systemic inflammation and oxidative stress—that drive both atherosclerotic progression and COPD pathogenesis [27]. These indicated COPD status may aid in identifying AMI patients at elevated risk for HF, enabling more tailored clinical management.

This study also has several limitations. First, the inherited shortcoming of retrospective study could not completely avoid selection bias. However, this dataset was derived from a multicenter and relatively large training set was used. Second, the model’s discriminative power, as measured by the C-index, was modest. This is likely a consequence of our intentional design choice to prioritize simplicity and immediate clinical applicability by excluding more complex predictors such as detailed laboratory biomarkers or echocardiographic parameters. However, we posit that the value of this nomogram lies in its ability to provide a practical and immediate risk assessment as discussed above. Finally, external validation in independent populations—particularly non-Chinese or Western cohorts—has not been performed, and the model’s generalizability across diverse ethnic and healthcare settings requires further evaluation. Notably, the CCC project is multicenter registry and its large sample size, appreciable geographic distribution and patients’ diversity endorses the generality of the nomogram.

## 5. Conclusions

Using six simple-to obtain parameters, we developed and validated a novel risk score to stratify the risk of in-hospital HF in AMI patients. This practical tool enables the early identification of high-risk individuals, facilitating timely interventions to improve clinical outcomes in such population.

## Figures and Tables

**Table 1:** Baseline characteristics of AMI patients with or without in-hospital HF.

Characteristic	Training Cohort			Validation Cohort		
	HF (n = 1467)	No HF (n = 35,881)	*p*	HF (n = 1867)	No HF (n = 38,159)	*p*
Age, means ± SD	68.72 ± 12.24	62.05 ± 12.41	<0.0001	68.91 ± 12.18	62.02 ± 12.39	<0.0001
Male, n (%)	978 (66.67%)	27,773 (77.40%)	<0.0001	1059 (66.52%)	27,624 (77.25%)	<0.0001
Clinical conditions at admission						
Heart rate	81.27 ± 17.11	76.54 ± 14.85	<0.0001	80.99 ± 17.80	76.69 ± 14.78	<0.0001
Systolic pressure	128.56 ± 24.18	130.82 ± 22.61	0.0002	127.50 ± 23.23	130.73 ± 22.69	<0.0001
Diastolic pressure	78.16 ± 15.76	78.94 ± 14.19	0.0405	77.34 ± 14.61	78.90 ± 14.10	<0.0001
Killip classification			<0.0001			<0.0001
I, n (%)	1054 (71.85%)	28,742 (80.10%)		1112 (69.85%)	28,360 (79.31%)	
II, n (%)	413 (28.15%)	7139 (19.90%)		480 (30.15%)	7397 (20.69%)	
Medical History						
Pre MI, n (%)	119 (8.11%)	2290 (6.38%)	0.0082	141 (8.86%)	2383 (6.66%)	0.0007
Pre PCI, n (%)	93 (6.34%)	2221 (6.19%)	0.8158	104 (6.53%)	2208 (6.18%)	0.5623
Pre CABG, n (%)	8 (0.55%)	131(0.37%)	0.2665	11 (0.69%)	129 (0.36%)	0.0349
AF, n (%)	65 (4.43%)	538 (1.50%)	<0.0001	59 (3.71%)	524 (1.47%)	<0.0001
CHF, n (%)	43 (2.93%)	261 (0.73%)	<0.0001	43 (2.70%)	269 (0.75%)	<0.0001
HTN, n (%)	822 (56.03%)	18,164 (50.62%)	<0.0001	901 (56.60%)	18,115 (50.66%)	<0.0001
DM, n (%)	398 (27.13%)	7458 (20.79%)	<0.0001	423 (26.57%)	7303 (20.42%)	<0.0001
Dyslipidemia, n (%)	96 (6.54%)	2516 (7.01%)	0.4908	93 (5.84%)	2585 (7.23%)	0.0357
Smoking, n (%)	510 (34.76%)	16,185 (45.11%)	<0.0001	583 (36.62%)	15,823 (44.25%)	<0.0001
Alcohol Use, n (%)	115 (7.84%)	2981 (8.31%)	0.5232	129 (8.10%)	2842(7.95%)	0.8231
Bleeding history, n (%)	24 (1.64%)	161 (0.45%)	<0.0001	17 (1.07%)	176 (0.49%)	0.0017
Stroke/TIA, n (%)	208 (14.18%)	2722 (7.59%)	<0.0001	210 (13.19%)	2758 (7.71%)	<0.0001
Peripheral VD, n (%)	21 (1.43%)	249 (0.69%)	0.0011	16 (1.01%)	259 (0.72%)	0.1999
COPD, n (%)	53 (3.61%)	375 (1.05%)	<0.0001	50 (3.14%)	383 (1.07%)	<0.0001
Renal dysfunction, n (%)	52 (3.54%)	417 (1.16%)	<0.0001	60 (3.77%)	384 (1.07%)	<0.0001
STEMI, n (%)	1115 (76.01%)	25,025 (69.74%)	<0.0001	1202 (75.50%)	25,094 (70.18%)	<0.0001
NSTEMI, n (%)	352 (23.99%)	10,856 (30.26%)	<0.0001	390 (24.50%)	10,663 (29.82%)	<0.0001

AF = atrial fibrillation; CABG = coronary artery bypass grafting; CHF = chronic heart failure; COPD = chronic obstructive pulmonary disease; DM = diabetes mellitus; HF = heart failure; HTN = hypertension; MI = myocardial infarction; NSTEMI = non-ST-segment elevation myocardial infarction; PCI = percutaneous coronary intervention; STEMI = ST-segment elevation myocardial infarction; TIA = transient ischemic attacks; VD= vascular disease.

**Table 2:** Results of multivariate logistic regression model.

Variables	Beta Coefficient	Odds Ratio [95% CI]	*p* Value
Age	0.0418	1.043 (1.038–1.047)	<0.0001
Heart rate	0.0177	1.018 (1.015–1.021)	<0.0001
Previous AF	0.4068	1.502 (1.124–1.975)	0.0046
Previous CHF	0.5949	1.813 (1.259–2.551)	0.0009
Previous COPD	0.7477	2.112 (1.545–2.832)	<0.0001
Previous CKD	0.7357	2.087 (1.520–2.809)	<0.0001

AF = atrial fibrillation; CHF = chronic heart failure; CKD = chronic renal dysfunction; COPD = chronic obstructive pulmonary disease; DM = diabetes mellitus.

**Table 3:** Heart Failure events by risk score categories.

Variables	Points	Total Score	Risk
Age	0–100	≤60 >60 and ≤70	low risk moderate risk
Heart rate	0–47		
Previous AF	0–10		
Previous CHF	0–14	>70 and ≤80	high risk
Previous COPD	0–18		
Previous CKD	0–18	>80	very high risk

AF = atrial fibrillation; CHF = chronic heart failure; CKD = chronic renal dysfunction; COPD = chronic obstructive pulmonary disease; DM = diabetes mellitus; HR = heart rate; SBP = systolic blood pressure.

## Data Availability

The data presented in this study are available on request to the corresponding author (ccc_csc@163.com or zhpdoc@126.com) for purposes of reproducing the results or replicating the procedure. The data are not publicly available due to privacy restrictions.

## Supplementary Materials

The following supporting information can be downloaded at: https://www.mdpi.com/article/10.3390/jcm15010194/s1.

## Abbreviations

The following abbreviations are used in this manuscript:

ACS	Acute coronary syndrome
AF	Atrial fibrillation
AMI	Acute myocardial infarction
CHD	coronary heart disease
CHF	Chronic heart failure
CKD	Chronic kidney disease
COPD	Chronic obstructive pulmonary disease
DBP	Diastolic blood pressure
DM	Diabetes mellitus
HF	Heart failure
HR	Heart rate
MI	myocardial infarction
NSTEMI	non-ST elevation myocardial infarction
OR	Odds ratios
SBP	systolic blood pressure
STEMI	ST elevation myocardial infarction
